# Supplementary material for: Absent Metabolic Transition from the Early to the Late Period in Non-Survivors Post Cardiac Surgery
Source: Nutrients. 2022 Aug 17;14(16):3366. doi: 10.3390/nu14163366 (PMC9416122; doi:10.3390/nu14163366)
Supplement: Supplementary file 1 [file nutrients-14-03366-s001.zip › nutrients-1842006-supplementary.pdf]

Absent metabolic transition from the early to the late period in non-survivors post cardiac surgery

Cecilia Veraar [1], Arabella Fischer [1], Martin H. Bernardi [1], Isabella Sulz [2], Mohamed Mouhieddine [1], Martin Dworschak [1], Edda Tschernko [1], Andrea Lassnigg [1] and Michael Hiesmayr [1,2]

[1] Department of Anesthesiology, Intensive Care Medicine and Pain Medicine, Division of Cardiac Thoracic Vascular Anesthesia and Intensive Care Medicine, Medical University of Vienna, 1090 Vienna, Austria
[2] Center for Medical Statistics, Institute for Medical Statistics, Informatics and Intelligent Systems, Medical University Vienna, 1090 Vienna, Austria
* Correspondence: cecilia.veraar@meduniwien.ac.at

Supplementary material

Supplementary Table S1: Details on statistically significant differences in REE, O_2ER, DO_2, VO_2, COO and SvO_2 between survivors and non-survivors were depicted below.

	Survivors	Non-survivors	p-value
	**median (IQR)**	**median (IQR)**	
**REE (**kcal/d)			
Day 0	1551 (1265, 1815)	1145 (629, 1577)	p<0.001
Day 1	1662 (1401, 1919)	1220 (828, 1525)	p<0.001
Day 2	1698 (1407, 1929)	1235 (893, 1523)	p<0.001
Day 3	1690 (1394; 1959)	1255 (973, 1519)	p<0.001
Day 4	1667 (1444, 1961)	1293 (979, 1533)	p<0.001
**O_2ER** (%)			
Day 2	31 (25, 34)	29 (24, 32)	p=0.018
Day 3	32 (27, 37)	29 (34, 32)	p=0.002
Day 4	33 (28, 39)	30 (24, 35)	p=0.018
Day 6	34 (29, 39)	29 (26, 33)	p=0.014
**DO_2** (ml/min)			
Day 0	786 (617, 933)	582 (431, 803)	p<0.001
Day 1	794 (674, 959)	617 (468, 760)	p<0.001
Day 2	774 (646, 914)	605 (514, 770)	p<0.001
Day 3	742 (630, 860)	625 (504, 842)	p<0.001
Day 4	736 (631, 843)	663 (535, 835)	p<0.001
**VO_2** (ml/min)			
Day 0	223 (182, 261)	164 (90, 226)	p<0.001
Day 1	239 (201, 276)	174 (119, 219)	p<0.001
Day 2	244 (202, 277)	177 (128, 219)	p<0.001
Day 3	243 (200, 281)	180 (140, 218)	p<0.001
Day 4	239 (207, 282)	186 (139, 220)	p<0.001
**COO (**l/min)			
Day 0	5.4 (4.3, 6.4)	4.2 (3.4, 5.8)	p<0.001
Day 1	4.7 (3.7, 5.9)	5.6 (4.6, 6.4)	p<0.001
Day 2	5.9 (4.8, 7.0)	4.7 (3.9, 6.3)	p<0.001
Day 3	5.8 (4.9, 6.8)	5.0 (4.0, 6.3)	p<0.001
Day 4	5.8 (4.8, 6.7)	5.3 (4.3, 6.4)	p<0.001
Day 5	5.4 (4.6, 6.4)	5.1 (4.1, 6.4)	p<0.001
**SvO_2**			
Day 1	68 (62, 73)	69 (64, 76)	p<0.001
Day 2	67 (61, 72)	69 (64, 74)	p<0.001
Day 3	65 (65; 71)	70 (65, 74)	p<0.001
Day 4	65 (59, 70)	69 (64, 74)	p<0.001
Day 5	65 (59, 70)	67 (62, 73)	p<0.001
Day 6	65 (60, 70)	69 (63, 72)	p<0.001
Day 7	65 (59, 71)	69 (64, 72)	p<0.001

CCO, continuous cardiac output; DO2, oxygen delivery; IQR, interquartile range; O2ER, oxygen extraction ratio, REE, resting energy expenditure; SvO2, mixed venous oxygen saturation; VO2, oxygen consumption
